# Rifaximin resistance in *Clostridioides difficile* is associated with specific *rpoB* alleles and multilocus sequence typing (MLST) clades

**DOI:** 10.1186/s12866-025-04164-4

**Published:** 2025-07-29

**Authors:** Julian Schwanbeck, Friederike Laukien, Thomas Riedel, Boyke Bunk, Philipp Halama, Cathrin Spröer, Jörg Overmann, Paul Cooper, R. Lia Kusumawati, Uwe Groß, Wolfgang Bohne, Andreas E. Zautner

**Affiliations:** 1 Institut Für Medizinische Mikrobiologie Und Virologie, Universitätsmedizin Göttingen, Göttingen, Germany; 2 Present Address: BioTechnology Institute, University of Minnesota-Twin Cities, St. Paul, MN USA; 3 Leibniz-Institut DSMZ-Deutsche Sammlung Von Mikroorganismen Und Zellkulturen GmbH, Brunswick, Germany; 4 Deutsches Zentrum für Infektionsforschung (DZIF) Partner site Hannover–Brunswick, Braunschweig, Germany; 5 St. Martin de Porres Hospital, Eikwe, W/R Ghana; 6 Department of Microbiology, Faculty of Medicine, Universitas Sumatera Utara, Medan, Indonesia; 7 Present address: Institute of Medical Microbiology and Hospital Hygiene, Medical Faculty, Otto Von Guericke University, Leipziger Str. 44, Magdeburg, 39120 Germany

**Keywords:** *Clostridioides difficile*, Nosocomial diarrhea, Antibiotic resistance, Rifaximin, Germany, Ghana, Indonesia, *rpoB*

## Abstract

**Background and Objectives:**

Rifaximin (RFX) has recently been suggested as an alternative treatment option for *Clostridioides difficile* infection. This study reports the survey on RFX susceptibility within a *C. difficile* test cohort that represents the five clinically relevant phylogenetic clades.

**Methods:**

Agar dilution assays were conducted to determine the minimum inhibitory concentrations (MICs) of RFX for 129 clinical *C. difficile* isolates from Germany (86), Indonesia (29), and Ghana (14). Genome sequence data were obtained for 50 representative isolates, including all those with a minimum inhibitory concentration MIC[RFX] of ≥ 32.0 µg/mL, to identify the underlying *rpoB* gene resistance alleles, determine the multilocus sequence typing (MLST) sequence types (STs), and infer phylogenetic relatedness.

**Results:**

10.1% of the isolates were found to be resistant to RFX. The resistance rate varies by region, with 4.7% in Germany, 27.6% in Indonesia, and 7.1% in Ghana. Three distinct *rpoB* alleles were associated with RFX resistance. The presence of a specific *rpoB* allele correlates with the MLST-based ST of the isolate, indicating that the rifaximin-resistant isolates belong to phylogenetic clades 1, 2, and 4. These isolates are represented by six different ribotypes: 010, 017, 027, 046, 084, and 131. Furthermore, we identified seven amino acid substitutions resulting from SNPs in the *rpoB* gene through alignment analysis. These substitutions are found in both RFX-resistant and susceptible isolates, suggesting that they are neutral mutations in relation to RFX susceptibility. These observations also indicate that RFX resistance arose independently in different clades.

**Conclusions:**

A substantial rate of RFX resistance, particularly among Indonesian isolates, was observed. This may be attributed to the prolonged use of rifampicin, especially in the treatment of tuberculosis. RFX resistance has been linked to specific amino acid substitutions in the β-subunit of RNA polymerase encoded by the *rpoB* gene. To the best of our knowledge, one of the identified RFX resistance-associated *rpoB* alleles (H502N, R505K, I750M) has not been previously described, whereupon, the amino acid substitutions I750M as well as I750V, E1037Q, A1205V, N1207A, A1208T, and D1232E were identified as neutral mutations that do not confer resistance to RFX.

**Supplementary Information:**

The online version contains supplementary material available at 10.1186/s12866-025-04164-4.

## Introduction

*Clostridioides difficile*, a Gram-positive, spore-forming bacterium, is the leading cause of nosocomial diarrhea worldwide and therefore a substantial burden to the healthcare systems [1, 2]. The incidence of infections with *C. difficile* (CDI) has increased within the last years in Germany and other, mainly western, countries [3, 4]. Most of the CDI occur after antibiotic exposure and are the foremost cause of hospital-acquired diarrhea. Typical antibiotics that reduce the microbiome to such an extent that a CDI occurs are clindamycin, ampicillin, amoxicillin, cephalosporins, and fluoroquinolones [5]. After antibiotic exposure and in combination with risk factors, CDI can lead to severe complications, like pseudomembranous colitis or toxic megacolon. Standard therapy of CDI includes the oral administration of fidaxomicin, vancomycin, or metronidazole [6]. However, the relapse rate after these primary therapy schemes is up to 20% [7]. In addition, vancomycin treatment, but also metronidazole treatment have been demonstrated to select vancomycin resistant enterococci (VRE) and thus leads to a growing infection prevention and control issue [8].

Rifaximin (RFX), a derivate of rifampicin, was recently proposed as a possible alternative therapy option for *C. difficile* infection (CDI), especially in cases of recurrent infections [9]. In several studies RFX was described as a follow-up therapy after an unsuccessful vancomycin therapy [10]. It is poorly absorbed in the gastrointestinal tract, making it suitable for non-systemic intra-intestinal use, similar to orally administered vancomycin [11]. Like rifampicin, RFX acts as an inhibitor of the bacterial RNA polymerase subunit encoded by the *rpoB* gene and thus inhibits bacterial transcription [12, 13]. RFX is licensed for the treatment of traveler’s diarrhea and hepatic encephalopathy in patients with liver cirrhosis in Germany, the United Kingdom and other European countries [14]. In these countries the treatment of the CDI with RFX is therefore still an off-label use. In contrast, RFX is also used for the treatment of pseudomembranous colitis due to *C. difficile*, small bowel bacterial overgrowth, irritable bowel syndrome and diverticulitis in other countries, such as the USA [14]. Promising treatment successes have already been observed in the treatment of recurrent CDI with RFX, but the use of RFX is not recommended in patients with previous exposure to any rifamycin due to the rapid development of resistance [10, 15–17]. The therapeutic application of rifaximin can lead to the swift emergence of resistance not only in *C. difficile* but also in other bacteria of the microbiome, potentially within just a few days of treatment, which significantly restricts its therapeutic indications [18–21]. The necessity for RFX/rifamycin susceptibility testing arises additionally from the widespread use of this drug class in veterinary medicine. Rifaximin is used for the prevention and treatment of mastitis in dairy cows [22]. Its low systemic absorption and low toxicity make it suitable for local therapy, minimizing drug residues in milk and reducing the risk of negatively impacting milk quality. Rifaximin is also administered intrauterinely for the treatment of endometritis in cattle and horses, and it is utilized for dermatological diseases in various animal species [23]. Furthermore, Rifampicin is primarily used in horses, for example, to treat *Rhodococcus equi* pneumonia [24], as well as in dogs and cats for infections caused by susceptible bacteria [25].

Several epidemiological surveys, along with a study by Pecavar and colleagues utilizing high-resolution melting analysis, have identified the following amino acid substitutions resulting from single nucleotide polymorphisms in the *rpoB* gene that are associated with reduced susceptibility to rifaximin in *C. difficile*: R505K, H502N + R505K, H502L, H502Y + L487F, H502N + A555A, R505K + I548M, D492Y, D492N, D492V, S488Y, S550Y, S475S + F481F + D492D + T501T + A508A + G510G + T539T + K556K + S575A, S507L, Q489L, G510R, and L584F [13, 16, 26, 27]. Among these, R505K is the most common mutation found in rifaximin-resistant isolates. Almost all of these mutations do not lead to a loss of in vitro fitness, with the exceptions of S507L, D492Y, and S550Y. This suggests that rifaximin resistance in *C. difficile* isolates may persist in clinical settings for an extended period [26].

The aim of this study was to perform a surveillance regarding RFX susceptibility in a *C. difficile* test cohort that covers the five clinically relevant clades out of eight established clades of this microbial species [28]. For this purpose, 129 clinical *C. difficile* isolates from Germany, Indonesia and Ghana were assessed using agar dilution susceptibility testing. At a MIC [RFX] ≥ 32.0 μg/mL, a resistance of the tested isolate to RFX was assumed. Whole genome sequencing was conducted on 50 isolates, including all isolates tested for RFX resistance, to assess the phylogenetic relatedness of the resistant isolates and to identify the underlying mutations in the *rpoB* gene, referencing the *rpoB* sequences deposited in the NCBI GenBank.

## Materials and methods

### Patient samples and isolate collection

A total of 129 clinical *C. difficile* isolates from Germany (86), Indonesia (29), and Ghana (14) were analyzed for their susceptibility to Rifaximin in this study. Among these isolates, 76 were derived from two previous studies: one conducted at St. Martin de Porres Hospital in Eikwe, Ghana [29], and the other at Adam Malik Hospital and Pematang Siantar Hospital in Medan, Indonesia, as well as at the University Medical Center Göttingen and Asklepios Hospital Schildautal in Seesen, Germany [30]. Whole-genome sequencing was performed for 47 of these isolates [29, 30]. To expand the basis of this study, an additional 53 patient isolates, collected during routine diagnostics at the University Medical Center Göttingen, were included; however, whole-genome sequencing data are not available for these additional isolates. Participants from the two studies in Ghana and Indonesia, from which *C. difficile* was isolated, were recruited between September 2013 and October 2014. They were classified into two groups: inpatients with diarrhea (the symptomatic group) and a non-diarrheic control group, which included patients, relatives of patients, or hospital staff without diarrhea. Notably, the isolates from the non-diarrheic control group were predominantly atoxigenic [29, 30]. The isolates from patients in Germany were collected between September 2013 and December 2017 and originated from inpatients whose samples were sent for diagnostic testing due to suspected CDI. According to the established stepwise diagnostic protocol, which includes screening by Glutamate Dehydrogenase (GDH) ELISA, specific detection of toxins A and B using PCR (Seegene GI-Bacteria(II) Assay, Seegene Inc., Seoul, Southkorea), and subsequent culture, these isolates are classified as toxigenic strains. The isolates were selected to ensure that no duplicate strains were included in the cohort, meaning there were no follow-up isolates from the same patient and no outbreak strains. If isolates were derived from the same patient, they represented different morphotypes.

Initial species confirmation was performed using the MALDI Biotyper system (Bruker Daltonics, Bremen, Germany). Results with MALDI Biotyper identification score values ≥ 2.000 were used as a threshold for the unambiguous identification of *C. difficile*. The isolates from these three geographic regions, particularly through the inclusion of the 76 ribotyped and partially genome-sequenced isolates, ensured that the test cohort exhibited high genetic diversity while also representing the five most clinically relevant and prevalent clades of this microbial species, as identified through ribotyping among the eight established clades. A complete list of the isolates can be found in Supplementary Material 3.

### PCR ribotyping and toxinotyping

The 76 isolates collected during the two previous studies conducted in Eikwe, Ghana; Medan, Indonesia; and Göttingen and Seesen, Germany [29, 30] were toxinotyped and PCR ribotyped using Bidet primers. This was done via agarose gel electrophoresis for the isolates from Ghana and Indonesia, and capillary gel electrophoresis for the isolates from Germany, following the consensus protocols established by *Clostridium difficile* Ribotyping Network (CDRN) and European *Clostridium difficile* Infection Surveillance Network (ECDIS-Net), as previously described [31–35].

### Bacterial culture of *C. difficile*

*C. difficile* strains were maintained in store as cryobank stocks (Mast Diagnostica, Reinfeld, Germany) at − 80 °C. For investigation they were thawed and grown at 37 °C on Columbia agar with 5% sheep blood (COS, bioMérieux, Germany). Freezing and thawing generally do not affect the susceptibility of *C. difficile* to most antimicrobials, including rifaximin. However, there are reports indicating that storage without selective pressure, such as freezing at −80 °C, can lead to the loss of plasmids (pCD-METRO) that confer resistance to metronidazole, resulting in a return to susceptibility to metronidazole [36]. Growth was performed either under anaerobic conditions using a COY anaerobic gas chamber (COY Laboratory Products, USA), with an atmosphere consisting of 85% N_2_, 10% H_2_, and 5% CO_2_, or alternatively in an Anaerocult® chamber (Merck, Darmstadt, Germany) using a GENbox anaer atmosphere generator (bioMérieux, Nürtingen, Germany).

### Antimicrobial resistance testing of *C. difficile*

Agar dilution susceptibility testing of the *C. difficile* isolates, regarded as the gold standard for resistance testing despite being somewhat time-consuming and resource-intensive, was conducted with slight modifications as described by Schwalbe and colleagues [37]. Every isolate was grown over night in 4 mL BHIS (37 g/L Brain Heart Infusion broth with 5 g/L yeast extract and 0.3 g/L cysteine). The bacterial cell suspension of each strain was suspended in BHIS to obtain a McFarland of 0.5 (corresponding to an OD_600_ of 0.1). The adjusted suspensions were diluted 1:10 in sterile saline and 2 µL were spotted on *Brucella* plates with log_2_ RFX concentrations between 0.008 µg/mL and 32 µg/mL (RFX was dissolved in methanol). Additionally, an antibiotic-free control plate was prepared simultaneously to ascertain overall strain growth, and on each plate a culture of *Bacteroides fragilis* ATCC 25285 was plated as a control for suitable anaerobic conditions. Furthermore, the reference strain *C. difficile* 630 ∆*erm* served as RFX susceptible control isolate. Inoculated plates were incubated for 48 h under anaerobic conditions at 37 °C and subsequently checked for bacterial growth (Supplementary Fig. 1).The procedure was carried out in technical duplicates and on three different occasions.

### Whole genome sequencing and bioinformatics

Whole genome sequencing was performed on the isolates that demonstrated resistance to Rifaximin, as previously described [38]. In short, the genomes of 47 *C. difficile* strains were sequenced on the PacBio *RSII* system (Pacific Biosciences, Menlo Park, CA, USA) using P6 chemistry. Genome assembly was accomplished applying the ‘RS_HGAP_Assembly.3’ protocol included in the SMRT Portal version 2.3.0. Long-read genome quality was enhanced by using the ‘RS_BridgeMapper.1’ protocol also included in the in SMRT Portal version 2.3.0. Further, a final genome sequence quality of QV60 was attained after mapping Illumina reads with the Burrows–Wheeler Aligner [39, 40] onto the genome sequence obtained by PacBio sequencing.

The genomes of the three German isolates (DSM105800—DSM105802) were sequenced using the HiFi technology on the PacBio Sequel *II*e system (Pacific Biosciences, Menlo Park, CA, USA). The assembly and error correction of these genomes were performed in SMRTLink version 13.0.0, utilising the “Microbial Assembly” and"Variant Calling"protocols, respectively. PacBio polishing with HiFi reads was conducted using pbmm2 and bcftools.

The obtained chromosomal contig of each strain was trimmed, circularized and adjusted to *dnaA* as the first gene. Multilocus sequence type (MLST), clade affiliation and toxin gene configuration were derived from the genome sequence and verified using the *Clostridioides difficile* MLST Database (https://pubmlst.org/cdifficile/) [41].

To infer the phylogeny of the RFX-resistant isolates, a core genome single-nucleotide polymorphism core SNP analysis was conducted using Snippy (https://github.com/tseemann/snippy) along with the genome of the reference strain *C. difficile* 630∆*erm*. A phylogenetic tree was constructed with RAxML-NG [42] using the GTR + G model with 1,000 bootstrap replications and was visualized using iTOL (http://itol.embl.de/) [43].

The *rpoB* protein sequences were extracted from the 50 sequenced high-quality genomes, aligned using the MAFFT aligner version 7.313 with default settings [44], and visualized the global alignment with Jalview 2.8 in a custom color scheme highlighting the mutations [45].

To extrapolate additional occurring mutations all 1,718 *rpoB* protein sequences of *C. difficile* were bulk extracted using NCBI's API (Entrez Direct) [46] and concatenated with the *rpoB* protein sequences of the high-quality sequenced genomes. Using a custom script, the dataset was filtered to retain only unique full-length sequences that were present in at least three genomes to disclose sequences with potential sequencing errors. These sequences were then aligned and visualized as described.

## Results

### Phenotypic rifaximin resistance testing of *C. difficile* isolates

**Fig. 1:**
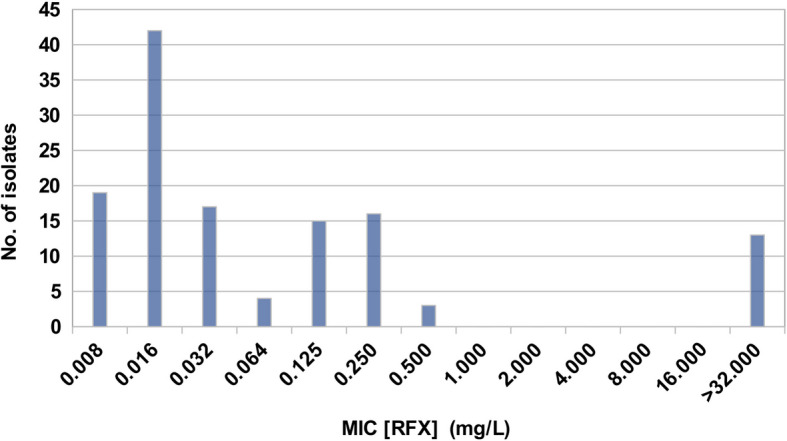
Frequency distribution of MIC [RFX]. The epidemiological cut off was determined at 0.5 mg/L, while RFX resistance was determined at an MIC ≥ 32 mg/L

Testing of the 129 *C. difficile* isolates yielded an epidemiological cut-off of RFX at a maximum MIC of 0.5 mg/L. As a result, 116 isolates demonstrated an MIC[RFX] ≤ 0.5 mg/L. The group of RFX resistant isolates clearly separates with a MIC ≥ 32 mg/L. The number of isolates at each MIC was 3 at 0.5 mg/L, 16 at 0.25 mg/L; 15 at 0.125 mg/L, 4 at 0.064 mg/L, 17 at 0.032 mg/L, 42 at 0.016 mg/L, 19 at 0.008 mg/L; and finally, 13 isolates at an MIC[RFX] ≥ 32.0 mg/L (Supplementary Material 3). The diagram of the frequency distribution of the MIC values for the tested *C. difficile* isolates (Fig. 1) shows a trimodal distribution with three local peaks at approximately 0.016 mg/L, 0.25 mg/L, and ≥ 32.0 mg/L. Furthermore, according to the frequency distribution, the MIC_50_ is 0.032 mg/L and the MIC_90_ is 32.0 mg/L. Accordingly, 89.9% (116/129) of the tested isolates were considered to be RFX susceptible and 10.1% (13/129) as RFX resistant. Analyzing the RFX resistance rate separately for the three countries included in the study, 4.7% (4/86) of the isolates from Germany, 27.6% (8/29) of the isolates from Indonesia and 7.1% (1/14) of the isolates from Ghana were tested resistant to RFX (Table 1). Thus, a significantly (p < 0.05) increased RFX resistance rate was observed for the Indonesian *C. difficile* isolates compared to the German isolates. There is a notable difference in the RFX resistance rates between the Indonesian isolates and the Ghanaian isolates, with rates of 27.6% and 7.1%, respectively. However, this difference is not statistically significant (p = 0.07) due to the relatively small number of *C. difficile* isolates available from Ghana.

**Table 1:** Total and RFX resistant number of strains per country. 129 Strains were collected from screenings in Germany, Indonesia and Ghana

Origin	No. of strains	RFX resistant (No.)	RFX resistant (%)
Germany	86	4	4.7
Indonesia	29	8	27.6
Ghana	14	1	7.1
Sum	129	13	10.0

### Epidemiological parameters associated with RFX resistance

The complete genomes of the 13 RFX-resistant *C*. *difficile* strains were sequenced, and from the resulting genome sequences, the MLST sequence type, clade, and ribotype were determined. Additionally, the toxin gene presence was investigated (Table 2). The RFX resistant *C. difficile* isolates collected in Germany belonged exclusively to MLST-ST 1 and thus to clade II. This is consistent with the information obtained from routine diagnostics using the Seegene GI-Bacteria(II) Assay, which indicates that the four German isolates are strains of the highly virulent ribotype 027. Consistent with this ribotype, both toxins A and B, as well as the genes for the *C. difficile* binary toxin (CDT), were detected. The sole RFX-resistant *C. difficile* isolate from Ghana is classified as MLST-ST 48, placing it within clade I. It has a ribotype of 084 but is non-toxigenic. The highest biodiversity among the RFX resistant isolates was found among the Indonesian isolates. This subgroup comprises five isolates that have an MLST-ST 37, categorising them within clade I and ribotype 017. As is typical for RT017 isolates, these five isolates possess only the toxin B gene, while testing negative for toxin A and CDT. Another clade IV isolate exhibited sequence type 39 and ribotype 131, and was found to be non-toxigenic. In addition, two of the Indonesian isolates were clade 1 isolates. However, these belonged to different sequence types and ribotypes. One isolate was classified as ST 35 and ribotype 046 and was tested positive for the toxin genes A and B. In contrast, the second Indonesian clade 1 isolate was classified as ST 15 and ribotype 010, which was found to be non-toxigenic, as is typical for RT 010 isolates.

**Table 2:** Epidemiological parameters and amino acid substitutions in the RpoB subunit of the RFX resistant (MIC[RFX] ≥ 32.0 µg/mL) tested *C. difficile* isolates compared to reference strain *C*. *difficile* 630 ∆*erm* (DSM 28645*,* CP016318.1)

DSM no	**Isolate ID**	**Origin**	**MLST-ST**	**RT**	**Clade**	**Toxins**	**NCBI GenBank** **Accession(s)**	**AA substitutions in RpoB**
DSM 29628	MC004-01–01	Indonesia	39	131	IV	-	CP016103	H502N, R505K, I750M^1^
DSM 29630	MC006-01–01	Indonesia	35	046	I	AB	CP016105	H502N, R505K
DSM 29640	MC016-01–01	Indonesia	15	010	I	-	CP016107	H502N, R505K
DSM 29646	MC022-01–01	Indonesia	37	017	IV	B	CP045167-CP045169	H502N, R505K, I750M^1^
DSM 29648	MC024-01–01	Indonesia	37	017	IV	B	CP045170-CP045171	H502N, R505K, I750M^1^
DSM 29695	MC010-01–01	Indonesia	37	017	IV	B	CP045173-CP045174	H502N, R505K, I750M^1^
DSM 29696	MC014-01–01	Indonesia	37	017	IV	B	CP169730-CP169732	H502N, R505K, I750M^1^
DSM 29697	MC019-01–01	Indonesia	37	017	IV	B	CP169727-CP169729	H502N, R505K, I750M^1^
DSM 100002	EH043-01–01	Ghana	48	084	IV	-	CP016099	H502N, R505K
DSM 28196^2^	6551/13	Germany	1	027	II	AB CDT	CP012320	R505K, I548M, E1037Q, A1205V, N1207A, A1208T, D1232E
DSM 105800^2^	1864/15	Germany	1	027	II	AB CDT	CP169726	R505K, I548M, E1037Q, A1205V, N1207A, A1208T, D1232E
DSM 105801^2^	2903/15	Germany	1	027	II	AB CDT	CP169725	R505K, I548M, E1037Q, A1205V, N1207A, A1208T, D1232E
DSM 105802^2^	3845/15	Germany	1	027	II	AB CDT	CP169724	R505K, I548M, E1037Q, A1205V, N1207A, A1208T, D1232E

^1^The AA substitution I750M was previously unpublished

^2^These substitutions are identical to those found in the R20291 type strain (CP029423.1, ribotype 027, clade 2)

**Fig. 2:**
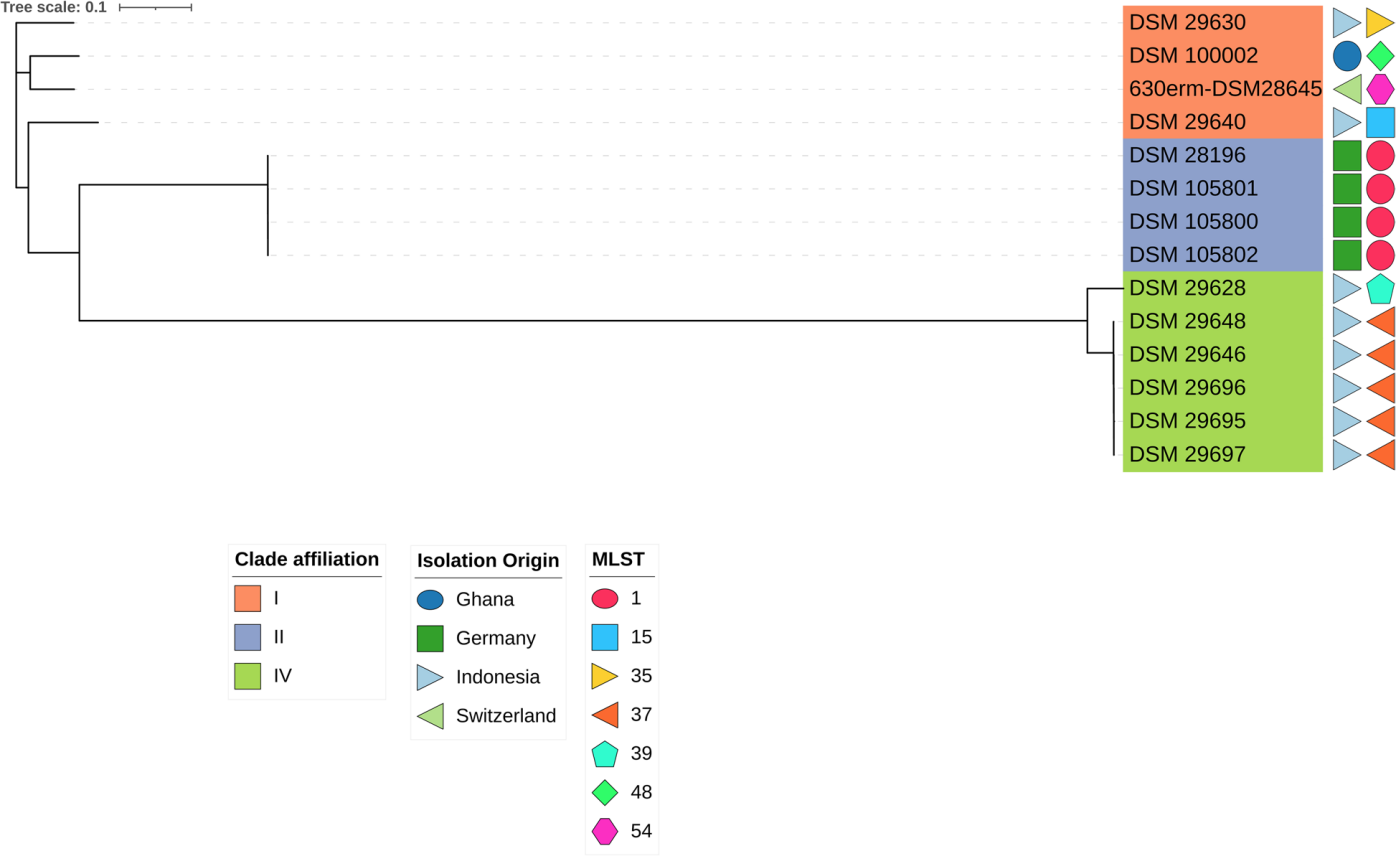
Midpoint-rooted phylogenetic tree of all detected RFX-resistant *C. difficile* isolates, based on GBDP distances calculated from genomic sequences. The branch lengths are scaled according to the GBDP distance formula d_5_. Numbers above the branches represent GBDP pseudo-bootstrap support values greater than 60% from 100 replications, with an average branch support of 75.3%. *C*. *difficile* 630 ∆*erm* (DSM 28645*,* CP016318.1) was inculded as reference in this analysis

A core genome single-nucleotide polymorphism-based phylogenetic analysis using Snippy (Fig. 2) clearly confirmed the classification of RFX-resistant isolates into clades I, II, and IV. Additionally, it shows the arrangement of four German isolates in MLST-ST 1 and five Indonesian isolates in MLST-ST 37. Complete Clonality could only be confirmed for the two isolates DSM105800 and DSM105802 from Germany.

### *rpoB* sequence polymorphisms in RFX resistant *C. difficile* isolates

To investigate *rpoB* sequence polymorphisms, we extracted the *rpoB* gene sequences from the genomes of the 50 sequenced isolates, which included the 13 RFX-resistant strains, and aligned them with the *rpoB* sequence of the reference strain *C. difficile* 630 ∆*erm* (DSM 28645, CP016318.1) [47]. In this dataset, which is limited to 50 study isolates, a total of 8 unique *rpoB* alleles were identified (Supplementary Material 3), including three different *rpoB* alleles within the 13 RFX-resistant strains (Table 2). In the four isolates from Germany, only one *rpoB* allele was identified. In comparison to the reference strain *C. difficile* 630 ∆*erm*, this allele was characterized by the amino acid substitutions R505K, I548M, E1037Q, A1205V, N1207A, A1208T, and D1232E. These substitutions are identical to those found in the ribotype 027, clade 2 type strain R20291 (GenBank accession: CP029423.1). The one RFX resistant isolate from Ghana, DSM 100002, was characterized by the amino acid substitutions H502N and R505K. Within the Indonesian RFX resistant strains, two isolates were also found that exhibited the latter two amino acid substitutions. In six, and thus the majority of the Indonesian strains, a further additional amino acid substitution, I750M, was detected, which was not found in the German isolates and the Ghanaian isolate. Looking at the *rpoB* alleles in relation to the clade grouping, a clear association between clades and *rpoB* alleles is evident in our test cohort. All RFX resistant clade 1 isolates carry the amino acid substitutions H502N and R505K, all clade 2 isolates the amino acid substitutions R505K, I548M, E1037Q, A1205V, N1207A, A1208T, and D1232E, and all clade 4 isolates the amino acid substitutions H502N, R505K, and I750M.

To assess whether the sample size was sufficient to detect a large difference in genotype frequency between RFX-resistant and RFX-susceptible isolates, a statistical power analysis was performed using the R function power.exact.test. By specifying an expected genotype prevalence of 80% in the resistant group (n = 13; R505K) and 10% in the susceptible group (n = 116), with a significance level of 0.05 and Fisher’s exact test as the method, the calculated statistical power was approximately 99% (i.e., > 0.99). This result demonstrates that the study is highly powered to detect such a pronounced difference between groups.

In our analysis of the *rpoB* alleles among RFX-susceptible isolates, we identified five additional alleles. First, we found that 23 isolates exhibit the wild-type (WT) allele of *C. difficile* 630 ∆*erm*. Additionally, 10 isolates carry the amino acid substitution I750M, while one isolate has an I750V substitution, and another isolate has a combination of the substitutions: E1037Q, A1205V, N1207A, A1208T, and D1232E. Finally, two isolates possess the V1143D amino acid substitution, which is associated with resistance to fidaxomicin [38].

For the alignment analysis of the *rpoB* sequences deposited in NCBI, we were able to include 1,629 full-length sequences based on our criteria, identifying 21 different *rpoB* alleles, i.e., those occurring at least three times (Supplementary Fig. 2). Of these, 40.58% (661/1,629) were found to be wild-type alleles, while the I750M polymorphism was detected in 26.46% (431/1,629) of the deposited *rpoB* sequences. The combination of I750M with H502N and/or R505K was detectable in 1.66% (27/1,629) of the recorded sequences.

Finally, no additional amino acid substitutions in the *rpoB* gene that could correlate with the two other local peaks at 0.016 mg/L and 0.25 mg/L of the trimodal distribution of the MIC values in the frequency distribution (Fig. 1) were identified.

## Discussion

One of the main findings from our study was the large difference in the RFX resistance rate between the Indonesian *C. difficile* isolates of 27.6% compared to the German (4.7%) and Ghanaian (7.1%) isolates. This increased RFX resistance rate among Indonesian isolates is mainly due to the high prevalence of RFX resistant isolates of ribotype 017 (ST 37). Of the 8 RFX-resistant isolates from Indonesia, 5 were assigned to ribotype 017. Our phylogenetic analysis showed that while these 5 isolates are related, they are not identical strains. In a recent East Asia-wide study by Lew and coworkers, it was shown that 67.7% of the ribotype 017 isolates were resistant for RFX [48]. Ribotype 017 is the most common *C. difficile* ribotype in Asia [49], and according to Lew and colleagues, RFX resistance is very widespread in East Asia, including Indonesian *C. difficile* isolates of ribotype 017. The exception here were ribotype 017 isolates from Australia, Japan and Singapore in which RFX resistance is rather rare [48]. While we were also able to detect RFX resistance in the ribotypes 010, 046, and 131 in Indonesia, Lew and colleagues occasionally detected RFX resistance in the ribotypes 002 and 018 [48]. The resistance rate to RFX also seems to be influenced by the age of the patients studied. For instance, in contrast to the Indonesian data, a recent study conducted at a tertiary pediatric hospital in Shanghai, China, reported a resistance rate of 0% for RFX and 2.0% for Rifampin [50].

Pecava and colleagues conducted a study with 348 *C. difficile* isolates from Austria, Slovenia and England in which Rifaximin resistance was also investigated [27]. In this study, 20.11% (70/348) of the examined *C. difficile* isolates proved to be RFX resistant. However, this was not a representative resistance study, as it investigated a pre-selected isolate collection, using strains from a previous study by Huhulescu and coworkers. In this study on 898 Austrian patient isolates only 7.46% (67/898) were tested as RFX resistant [11]. In our study, which is more representative of the clinical average in the sub-cohort of German *C. difficile* isolates and which to our knowledge is the first of this kind in Germany, we found a comparatively low RFX resistance rate of 4.65%. On the other hand, Reigadas and colleagues have conducted a study on phenotypic RFX resistance in Spain that examined 276 *C. difficile* isolates. There, 32.2% (89/276) of all isolates were tested RFX resistant, which is also significantly higher than we determined [51]. A similar study from the USA, conducted by Curry and colleagues, identified RFX resistance in 36.8% (173/470) of *C. difficile* isolates in a large American teaching hospital [16].

In our study, as well as in the studies conducted by Pacavar et al., O’Connor et al., and Curry et al., the majority of the RFX-resistant isolates were classified as ribotype 027 (North American pulsed-field type 1 = NAP1) and MLST-ST1, which is typical for *C. difficile* isolate cohorts from Central Europe and the United States [13, 16, 27]. *C. difficile* isolates of MLST-ST1 have also been associated with a 60% resistance rate to RFX in Korea [52]. In contrast, the majority of RFX resistant isolates in Spain were classified as ribotype 001 [51].

In the study of Pecavar et al. 13, and in the study of Curry et al. 5 different *rpoB* alleles were associated with RFX resistance. R505K was the most frequent amino acid substitution in RpoB associated with RFX resistance [16, 27]. This is consistent with the US-based study by O’Connor and colleagues, which found that in 14 rifaximin-resistant isolates (out of 80 tested), the R505K amino acid substitution was present in all resistant isolates, either as the sole substitution or in combination with S488T, H502N, or I548M [13]. Among the Ghanaian isolates, we identified only one RFX-resistant isolate, which was classified as ribotype 084. Like the other clade 1 isolates of ribotype 010 and 046 cultivated in Indonesia, it carries the RpoB amino acid substitutions H502N and R505K. These amino acid substitutions have already been described earlier, but the assignment to these somewhat rarer ribotype had not been made before.

In our study, all RFX-nonsusceptible isolates also displayed the R505K amino acid substitution. In addition, some further amino acid substitutions were identified in comparison to the reference strain allele. One of them, the amino acid substitution I750M in the Indonesian isolates of ribotype 017 was described here for the first time, to the best of our knowledge. It should be noted that these *rpoB* alleles are only associated with phenotypic RFX resistance while the exact mechanism of RFX resistance in *C. difficile* was not been fully elucidated, though antibiotic interaction with the β-subunit and the γ-subunit of bacterial RNA polymerase had been suspected [12]. In the context of our study, we analyzed the *rpoB* alleles from the genomes of 50 isolates, including 13 RFX-resistant and 37 RFX-sensitive isolates. We identified the amino acid substitutions I750M and I750V individually, as well as the combination of the substitutions E1037Q, A1205V, N1207A, A1208T, and D1232E in susceptible isolates. Additionally, both isolates with the V1143D amino acid substitution, which confers resistance to fidaxomicin [38], were also found to be RFX-susceptible. When considering the phenotypic MIC[RFX] values, these mutations can be classified as neutral mutations regarding RFX susceptibility. Thus, in our test cohort, the amino acid substitution R505K in combination with H502N or I548M primarily accounts for the RFX resistance mutation. The presence of RFX susceptibility-neutral mutations suggests that the amino acid substitutions conferring resistance arise under evolutionary pressure, indicating that RFX therapy is exerting selective pressure independently in different clades or strains. The limitations of analyzing only 50 whole genomes, along with the uneven representation of countries and strain types, are considerable. This significantly hinders our ability to identify additional neutral mutations or amino acid substitutions. Furthermore, the presence of at least 21 different alleles in the 1,629 full-length *rpoB* sequences deposited in NCBI GenBank demonstrates the high evolutionary variability at this gene locus, with 1.66% of the genomes exhibiting the amino acid substitutions I750M combined with H502N and/or R505K.

As Curry and colleagues have already shown on a small series of 8 patients, therapy with a rifamycin antibiotic, typically rifampicin, leads to a selection of RFX and rifampicin-resistant *C. difficile* strains [16]. This is very plausible since the underlying resistance mechanisms are identical [12]. As a result, there is typically cross-resistance between rifampicin and rifaximin [13]. This was further confirmed by the fact that rifampicin and RFX MICs correspond to each other and thus RFX susceptibility can be derived from rifampicin susceptibility [13, 51]. Since Indonesia is one of the top three tuberculosis burden countries together with India and China [53] and rifampicin remains a standard therapy for tuberculosis [54], it seems only plausible that in East Asia and especially in Indonesia there are very high rates of RFX resistance in *C. difficile* isolates. In this context, a 2013 outbreak of *C. difficile* infection among tuberculosis patients in Poland was linked to a highly rifampicin-resistant PCR ribotype 046 clone [55]. Our study also detected RFX resistance in isolates of ribotype 046. A recent study indicates that resistance genes and mutations conferring resistance to vancomycin, fidaxomicin, or metronidazole in patient isolates are, overall, relatively rare and are likely declining due to the intentional use of these antibiotics as part of Antibiotic Stewardship programs [56]. Regrettably, genomic data on RFX resistance determinants over time is currently unavailable. Two studies from Taiwan, one conducted in 2012 (with isolates from 2005 to 2010) and another in 2024 (with isolates from 2019 to 2021), indicate a decrease in the RFX resistance rate based on phenotypic data, from 10.9% to 2.6% [57, 58].

It is important to note that reduced susceptibility to RFX is also associated with specific strain types. Higher rates of rifampicin resistance have been observed in *C. difficile* ribotypes RT027 and RT017, primarily due to the unique genetic backgrounds of these strains, which facilitate the acquisition and persistence of mutations that confer resistance, particularly in the *rpoB* gene. Multiple studies have shown that these ribotypes exhibit an increased propensity for rifampicin resistance, with RT027 showing elevated resistance rates and RT017 demonstrating even higher rates, especially in hospital settings and certain geographic regions such as Asia [59–61]. In our study, RT027 and RT017 were also the dominant ribotypes associated with RFX resistance.

## Conclusion

The overall RFX resistance rate in our test cohort was 10.08%, largely due to the significant increased RFX resistant rate of Indonesian *C. difficile* isolates of 27.59%. This may be a consequence of the use of rifampicin, e.g. in the treatment of tuberculosis in Indonesia. Our study, which examines not only the RFX resistance rates but also the associated RpoB amino acid substitutions in isolates from Indonesia, Germany, and Ghana, provides new epidemiological data in this regard, as comparable studies have so far been conducted only in the USA, Canada, the UK, Austria, and Slovenia [13, 16, 27]. We were able to identify three different *rpoB* alleles in the tested strains associated with phenotypic RFX resistance. One of these alleles characterized by the three amino acid substitutions H502N, R505K, and I750M compared to the *rpoB* sequence of the reference strain *C. difficile* 630 ∆*erm* was previously unpublished. Through *rpoB* sequencing and comparison of RFX-susceptible and resistant isolates, an epidemiological association was established between the amino acid substitution I750M and RFX resistant isolates of clade IV. However, the detection of amino acid substitutions I750M, I750V, E1037Q, A1205V, N1207A, A1208T, and D1232E in susceptible isolates revealed that these substitutions are neutral mutations with respect to RFX susceptibility. Such *rpoB* mutations occur relatively quickly during therapy, and since they generally do not lead to a fitness loss, they can persist for a long time even after treatment has ended [26].

This underscores the necessity for current resistance surveillance, particularly regarding the phenotypic susceptibility to rifaximin. Surveillance based on molecular mechanisms, particularly *rpoB* mutations, remains challenging because these mechanisms have not yet been fully described.

Rifaximin, which remains in the intestinal lumen and is not absorbed, is still an alternative therapy option for *C. difficile* infection (CDI) in regions with low rifamycin usage rates or after confirming susceptibility. Therefore, before prescribing Rifaximin (RFX) for the treatment of CDI, it is important to obtain a travel history regarding East Asia and to exclude any previous treatments with Rifaximin or Rifampicin in the patient's medical record.

## Supplementary Information

Supplementary Material 1: Figure S1. Scheme of the Antimicrobial Susceptibility Testing workflow.

Supplementary Material 2: Figure S2. Aligment of unique full-length *rpoB* sequences obtained from NCBI GenBank.

Supplementary Material 3: List of all bacterial isolates included in the study.

## Data Availability

The genome sequence datasets generated and analyzed for this study have been deposited in NCBI GenBank and all bacterial strains have been deposited at the Leibniz-Institute DSMZ (Table 2). NCBI GenBank Accession(s): CP016103, CP016105, CP016107, CP045167-CP045169, CP045170-CP045171, CP045173-CP045174, CP169730-CP169732, CP169727-CP169729, CP016099, CP012320, CP169726, CP169725, CP169724.

## Abbreviations

AA: Amino acid
ATCC: American Type Culture Collection
BHIS: Brain Heart Infusion Supplemented
CDI: *C. difficile* Infection
COS: Columbia agar supplemented with sheep blood
DSMZ: Leibniz Institute DSMZ-German Collection of Microorganisms and Cell Cultures
*erm*: Erythromycin ribosome methylation gene
MAFFT: Multiple Alignment using Fast Fourier Transform
MALDI: Matrix-Assisted Laser Desorption/Ionization
MIC: Minimum inhibitory concentration
MLST: Multilocus sequence typing
NCBI: National Center for Biotechnology Information
RFX: Rifaximin
*rpoB*: RNA polymerase subunit beta gene
SNPs: Single-nucleotide polymorphism
ST: Sequence type
TYGS: Type (Strain) Genome Server
VRE: Vancomycin resistant *Enterococcus faecium*
WT: Wild-type
