# Supplementary figures and images for: Rifaximin resistance in Clostridioides difficile is associated with specific rpoB alleles and multilocus sequence typing (MLST) clades
Source: BMC Microbiol. 2025 Jul 29;25:458. doi: 10.1186/s12866-025-04164-4 (PMC12306076; doi:10.1186/s12866-025-04164-4)

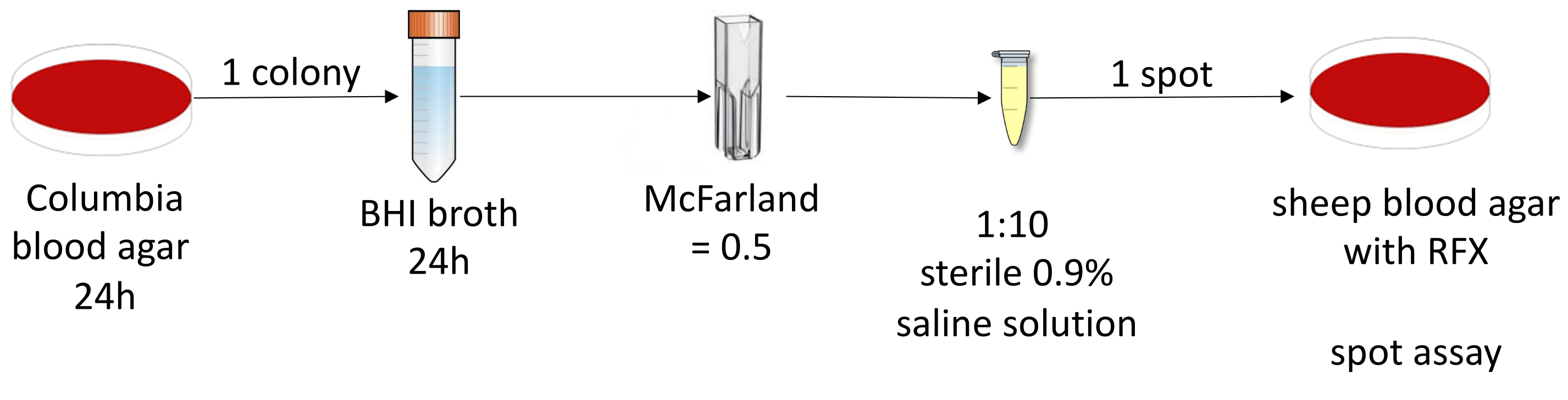

Supplement: Supplementary file 1 — Supplementary Material 1: Figure S1. Scheme of the Antimicrobial Susceptibility Testing workflow. [file 12866_2025_4164_MOESM1_ESM.jpg]
